# Rare-metal-free high-performance Ga*-*Sn-O thin film transistor

**DOI:** 10.1038/srep44326

**Published:** 2017-03-14

**Authors:** Tokiyoshi Matsuda, Kenta Umeda, Yuta Kato, Daiki Nishimoto, Mamoru Furuta, Mutsumi Kimura

**Affiliations:** 1 Innovative Materials and Processing Research Center, Ryukoku University, Japan HRC205, 1-5, Seta Oe-cho, Otsu, Shiga, 520-2194, Japan; 2 Department of Electronics and Informatics, Faculty of Science and Technology, Ryukoku University, Japan; 3 School of Environmental Science and Engineering, Kochi University of Technology, Japan; 4 Joint Research Centre for Science and Technology, Ryukoku University, Japan

## Abstract

Oxide semiconductors have been investigated as channel layers for thin film transistors (TFTs) which enable next-generation devices such as high-resolution liquid crystal displays (LCDs), organic light emitting diode (OLED) displays, flexible electronics, and innovative devices. Here, high-performance and stable Ga-Sn-O (GTO) TFTs were demonstrated for the first time without the use of rare metals such as In. The GTO thin films were deposited using radiofrequency (RF) magnetron sputtering. A high field effect mobility of 25.6 cm^2^/Vs was achieved, because the orbital structure of Sn was similar to that of In. The stability of the GTO TFTs was examined under bias, temperature, and light illumination conditions. The electrical behaviour of the GTO TFTs was more stable than that of In-Ga-Zn-O (IGZO) TFTs, which was attributed to the elimination of weak Zn-O bonds.

Oxide semiconductor materials such as In-Ga-Zn-O (IGZO)^1^,^2^,^3^,^4^, In-Sn-Zn-O (ITZO)^5^,^6^, In-Ga-O (IGO)^7^, and In-Sn-O (ITO)^8^ have several advantages as active layer of thin film transistors (TFTs) such as steep subthreshold swing (*S* factor), transparency, and extremely low leak current in off state when compared to conventional semiconductors such as hydrogenated amorphous silicon (a-Si:H)^9^ and polycrystalline silicon^10^. In addition, oxide TFTs have high field effect mobility (*μ*_FE_) and can be easily fabricated on large-area substrates by deposition processes with low cost, low toxicity, and low risk of explosion, such as radiofrequency (RF) or direct current (DC) magnetron sputtering. These techniques are used in the TFT fabrication processes to deposit IGZO as semiconductor, ITO as the transparent electrodes for displays, and metallic materials (Cr, Mo, and Al) for electrodes. The fabrication processes for oxide semiconductors can be easily integrated into or replace conventional TFT processes, such as photolithography and etching. Hence, not only information displays but also novel devices have been proposed such as memories^11^, processors, and other electronic elements^12^. However, IGZO, ITZO, IGO, and ITO include In in the matrix which is associated with high fabrication costs as In is a rare metal with few mining sites^13^ and is hence expensive. On the other hand, the In-free oxide semiconductor materials such as ZnO^14^,^15^,^16^,^17^,^18^, Al-Zn-Sn-O (AZTO)^19^, Zn-Sn-O (ZTO)^20^, ZnON^21^ include Zn, which is thought to be the reason of the instability of devices with forming the weak Zn-O bond^22^. The electrical characteristics and those stability of In-free TFTs were not enough for application to the novel devices. In addition, multi-metallic materials (especially IGZO) are difficult to mass produce because the stoichiometry of the deposited films can be different to that of the sputtering target due to preferential sputtering of some elements^2^. Sputtering targets can be of poor quality due to low crystallinity or incomplete oxidation of compounds^23^, which may reduce device performance. Furthermore, there are many defects in the semiconductor films^4^,^24^,^25^,^26^,^27^,^28^,^29^,^30^,^31^, and it is difficult to control the density of oxygen vacancies induced during the deposition^32^,^33^,^34^. Therefore, new semiconductor materials that do not use rare metals and consist of fewer elements are being explored.

In this paper, we first report a stable TFT with a high *μ*_FE_ that incorporate Ga-Sn-O (GTO) as the amorphous and transparent oxide semiconductor. This material is a dual metallic oxide without rare metals such as In. The stability of the GTO TFT was evaluated under various stress conditions and compared to that of IGZO TFTs.

## Results and Discussion

### Abundance in the Earth’s upper continental crust

**Figure 1:**
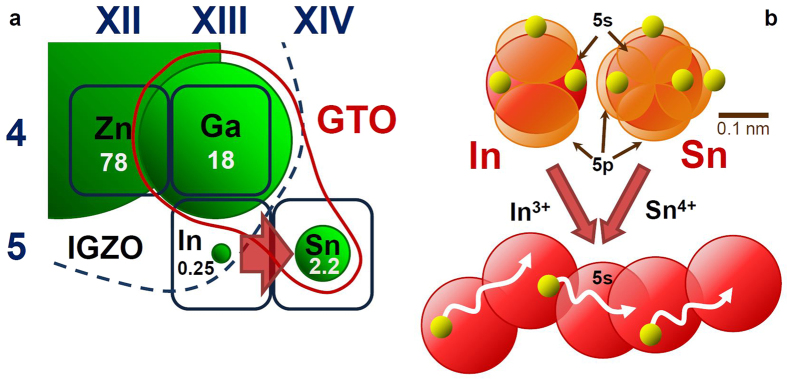
Subset of the periodic table of elements showing metallic elements of interest to oxide semiconductors, electron orbital of In and Sn in ground state^35^35 , and current path formed by metallic ions. (**a**) The relative abundance of the elements in the Earth’s upper continental crust is indicated by the green circles and the absolute values (ppm) are shown^13^. Ga and Sn as metallic elements of GTO are surrounded by red line, and In, Ga, Zn, elements of IGZO are surrounded by dashed blue line. (**b**) 5 s orbitals are indicated by red circles, 5p orbitals are indicated by orange ovals, electrons are indicated by yellow particles. In^3+^ ions and Sn^4+^ ions have the same electronic structure, and the path of electrons in IGZO and GTO can be formed by 5 s orbital without electrons described with red circles.

The abundance of Ga and Sn in the Earth’s upper continental crust is 18 ppm and 2.2 ppm, respectively, much higher than that of In (0.25 ppm)^13^, as shown in Fig. 1a. Therefore, substituting Sn for In is a reasonable countermeasure for avoiding the use of rare earth metals. Moreover, In and Sn have similar electronic structures and electrical properties. The high *μ*_FE_ observed for amorphous IGZO TFTs has been attributed to the electron path formed by the broad In 5 *s* orbital. In with atomic number 49 has an electronic structure in ground state; 1s^2^2s^2^2p^6^3s^2^3p^6^3d^10^4s^2^4p^6^4d^10^5s^2^5p. On the other hand, Sn with atomic number 50, the next element after In in the periodic table, has a similar electronic structure in ground state; 1s^2^2s^2^2p^6^3s^2^3p^6^3d^10^4s^2^4p^6^4d^10^5s^2^5p^2^ as shown in Fig. 1b^35^. As a result, In^3+^ ions in IGZO and Sn^4+^ ions in GTO have the same electronic structure of 1s^2^2s^2^2p^6^3s^2^3p^6^3d^10^4s^2^4p^6^4d^10^. Therefore, a high *μ*_FE_ is expected for GTO TFTs because the 5 s orbital of Sn serves as a path for current flow, similar to the 5 s orbital of In as shown in Fig. 1b^1^.

### Electrical characteristic of the GTO TFT

**Figure 2:**
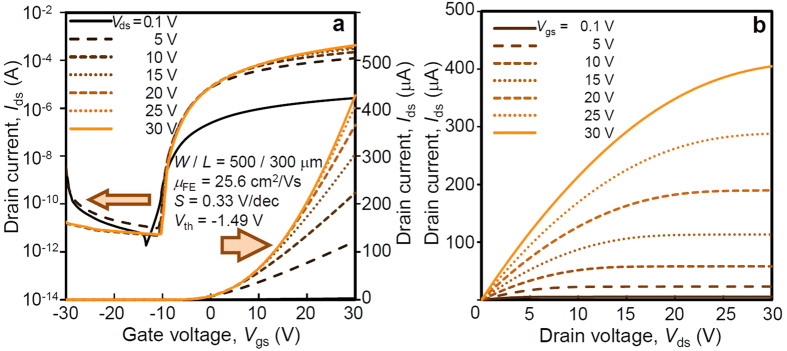
Electrical characteristics of the GTO thin film transistor. (**a**), transfer characteristics (drain current vs. gate voltage for various drain voltages) with left axis in log scale and right axis in linear scale, respectively, and (**b**) output characteristics (drain current vs. drain voltage for various gate voltages) in linear scale. The electrical characteristics of GTO TFT was excellent with low off current in negative gate voltage region in transfer characteristics, and high on current in positive gate voltage. The saturation characteristics in high drain voltage were clearly observed in output characteristics.

Figure 2 shows the drain current (*I*_ds_) as a function of the gate voltage (*V*_gs_) from −30 to 30 V with a 0.1 V step, and fixed drain voltage (*V*_ds_) of 0.1 V, from 5 to 30 V with a 5 V step. The ratio between the on current of the TFT (*I*_on_) at *V*_gs_ = 30 V and the off current (*I*_off_) at *V*_gs_ = −10 V, (*I*_on_/*I*_off_) was over 10^8^. The threshold voltage, *V*_th_ = −1.49 V, subthreshold swing, *S* = 0.33 V/dec where *S = *d*V*_gs_/dlog*I*_ds_, and the highest *μ*_FE_ of 25.6 cm^2^/V·s, were achieved for the GTO TFT in linear region at *V*_gs_ = 29.5 V and *V*_ds_ = 0.1 V, which values were comparable to those observed for IGZO TFTs^36^ and ITZO TFTs^5^,^6^. In the saturation region, the value of *μ*_FE_ was slightly lower than that of linear region. High *μ*_FE_ of linear region would be due to the path of electron formed in the conduction band at high *V*_gs_ and low *V*_ds_, whereas lower *μ*_FE_ of saturation region would be because the route of electron was partially formed by percolation path in pinch off region at high *V*_ds_. In addition, the saturation characteristics of GTO TFTs were excellent, and the TFT characteristics did not deteriorate, even after measurement at high voltages of *V*_gs_ = 30 V and *V*_ds_ = 30 V, as shown in Fig. 2b. The abovementioned excellent performances show that the GTO TFTs are suitable for use in next-generation displays such as 8 K ultra-high-definition televisions (UHDTV), LCD, and OLED^37^. The other GTO TFTs with different composition ratio from Ga: Sn = 1: 3 were fabricated, however, the best TFT performance was achieved with the current composition ratio.

### Optical absorbance and XRD intensity of the GTO film

**Figure 3:**
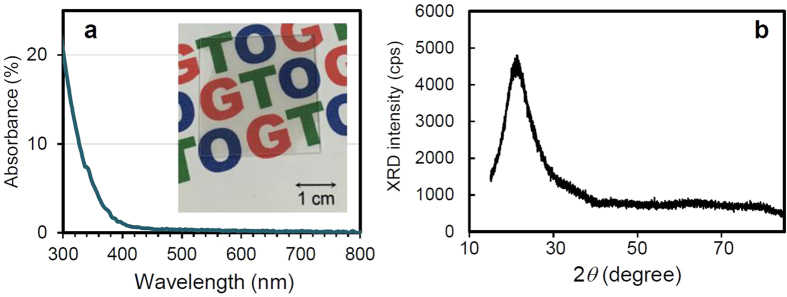
Optical absorbance and XRD pattern of the GTO film. (**a**) optical absorbance as a function of wavelength. The inset is a photograph of a GTO thin film deposited on a quartz glass substrate put on the top of a printed paper, showing the transparency of the thin film. (**b**) XRD pattern showing amorphous structure of GTO thin film deposited on a quartz substrate.

Figure 3a shows the optical absorbance of a GTO thin film deposited on a quartz substrate. The inset shows a photograph of the sample laid over printed text in red, green, and blue, showing the good transparency of the film in the visible wavelength range. The absorbance of the GTO thin film was lower than 20% at a wavelength of 300 nm and approximately zero above 380 nm. Figure 3b shows the XRD pattern of GTO thin film deposited on a quartz substrate. The structure of GTO thin film is amorphous, because the no diffraction peak was observed in the XRD pattern.

### Characteristic stability of the GTO TFT

A major drawback of conventional amorphous oxide semiconductors is the poor stability of TFTs under the driving conditions of devices^38^. In order to investigate the stability of the GTO TFTs, we performed accelerated operating tests such as the positive bias stress (PBS), positive bias temperature stress (PBTS), positive bias illumination stress (PBIS), negative bias stress (NBS), negative bias temperature stress (NBTS), and negative bias illumination stress (NBIS) tests.

**Figure 4:**
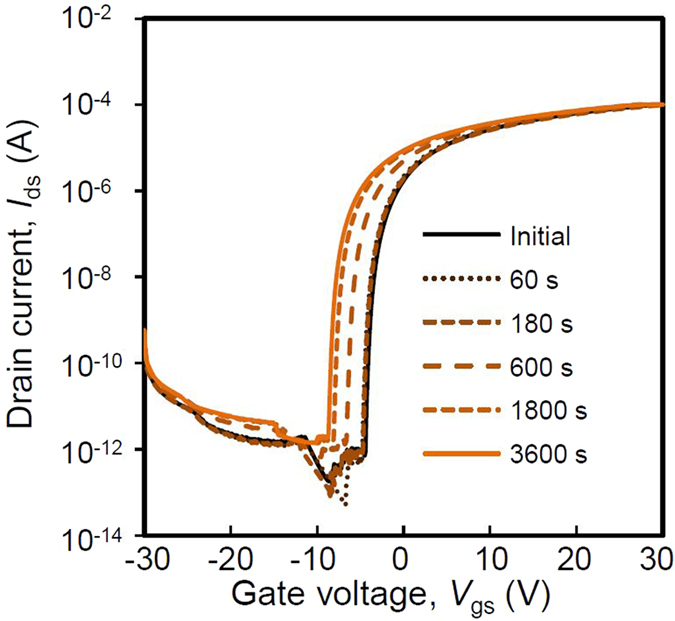
Negative bias illumination stress characteristics of the GTO TFT. The drain current is shown as a function of the gate voltage for various times up to 3600 s. The shift of electrical characteristics under the NBIS was parallel, and, therefore, the degradation of subthreshold swing was not observed.

Figure 4 shows the *I*_ds_*-V*_gs_ characteristics of a GTO TFT under the NBIS test conditions. Generally, amorphous oxide semiconductor TFTs have poor *I*_ds_*-V*_gs_ behaviour under NBIS testing^36^. However, only a negative shift of 4.3 V in *V*_gs_ was observed after applying NBIS conditions of a gate voltage of −20 V under light illumination for 3600 s. Excellent TFT characteristics were maintained even after the NBIS tests, such as low *I*_off_ values in the negative *V*_*gs*_ region and high *I*_on_ values in the high *V*_gs_ region. In addition, no degradation in the *S* factor was observed in the subthreshold region of *V*_gs_ from around −10 V to 5 V after the NBIS test. Therefore, we can conclude that the no trap state was generated close to the conduction band and NBIS effects were due to the capturing of fixed charges in the insulator, similar to the reported effect for IGZO TFT^38^. This effect is different from the hypothesis such as elimination of dangling bonds with O_2_ annealing^39^, and defect formation in semiconductor layer or interface between the semiconductor and gate insulator^40^,^41^,^42^. The shift in the electrical characteristics is due to a similar mechanism to that known for IGZO TFTs; holes from electron-hole pairs excited by the light and heat are transported from the back channel of the semiconductor to the gate surface and are captured close to the interface between the gate insulator and the semiconductor^38^.

**Figure 5:**
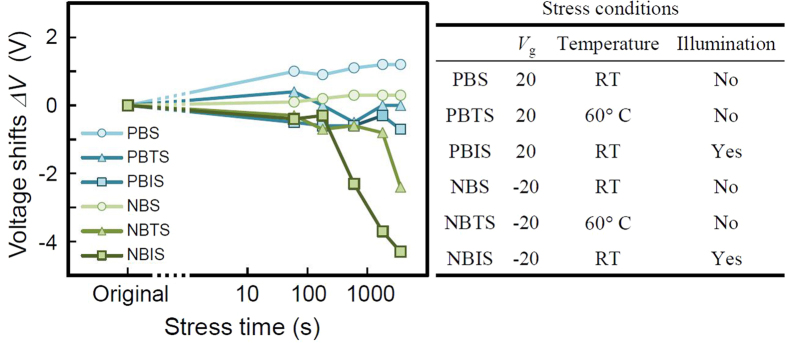
Results of various stress tests on the GTO TFT. Shifts in the turn-on voltage, which is defined as *V*_gs_ for *I*_ds = _1 × 10^−9^. A at the subthreshold region, (*ΔV*) at the subthreshold region are shown as a function of stress time under conditions of positive bias stress (PBS), positive bias temperature stress (PBTS), positive bias illumination stress (PBIS), negative bias stress (NBS), negative bias temperature stress (NBTS), and negative bias illumination stress (NBIS). The original state refers to the conditions before the stress test. The shift of the GTO TFT characteristics was largest for NBIS test.

Figure 5 shows the stability of GTO TFT under the PBS, PBTS, PBIS, NBS, NBTS, and NBIS tests without passivation. The stress conditions are shown in the table in Fig. 5. The shifts of the turn-on voltage, which is defined as *V*_gs_ for *I*_ds_ = 1 × 10^−9^ A, (*ΔV*) of all stress tests were less than that of NBIS tests. These results for NBTS and NBIS testing of GTO are better than those for IGZO TFTs, where the shift was over 10 V in the negative direction at 1000 s^36^. Therefore, it can be generally concluded that the stability of TFTs fabricated using GTO is better than that observed for IGZO. It was reported in the literature that weak bonds were formed between the Zn and O in IGZO^22^. As GTO does not include Zn in the matrix, we expect that the improved stability of the GTO TFT may be due to the absence of such weak bonds, because the enthalpy (heat) of formation of ZnO is smaller than Ga_2_O_3_ and SnO_2_, which means that the bonds in GTO is more stable than ZnO in IGZO. Therefore, the GTO lattice is expected to be stable during plasma processes, such as sputtering and CVD, for deposition of passivation films and dry etching process. In addition, the optical band gap of a GTO film obtained from a Tauc plot was 3.12 eV, which is larger than silicon-based semiconductors, and comparable to other oxide semiconductors, such as IGZO, ZnO, SnO_2_ and Ga_2_O_3_. This means that the generation of holes is limited. Hence, highly stable TFT characteristics are expected for GTO TFTs, because the large band gap means that the number of holes generated by light illumination is limited; the holes are the main reason for the NBIS instability of oxide semiconductor TFTs^38^. Moreover, the GTO is amorphous, and hence, large area uniformity is expected during the device fabrication process, because the amorphous oxide materials are thought to be more stable against grain boundaries formed in crystalline oxide semiconductors. The path of electrons in the GTO thin film is formed by the 5 s orbital of Sn ions, which is largely spreading in the matrix as shown in Fig. 1b. The uniform shape of the 5 s orbital maintains the current path in random direction of the bond between the elements in amorphous GTO matrix which was confirmed by XRD pattern. The role of Ga in GTO is similar to Ga and Zn in IGZO, which stabilize the amorphous structure with Coulomb potential. Zn stabilizes the amorphous structure in IGZO, however, Zn element is not needed in GTO for stabilizing the amorphous GTO TFT characteristics. In addition, improvement in the *μ*_FE_ and stability of GTO TFTs can be expected by appropriate passivation or treatment of the back channel and optimization of the fabrication process applied for IGZO TFTs^25^,^43^,^44^.

## Conclusion

High field effect mobility TFT with a low *S* factor was prepared using GTO, where the rare earth In was replaced by Sn. The stability of the GTO TFT without a passivation film under various accelerated operating conditions was significantly higher than that of equivalent IGZO TFTs. Although we compared the IGZO and GTO TFTs fabricated in our laboratory just for evaluation of the materials themselves, because TFT characteristics are influenced by the treatment of backchannel such as passivation materials and methods^25^,^44^,^45^ and surface treatment^36^,^43^, the effective treatments for IGZO TFT can also be effective for GTO TFT. We propose such GTO TFTs as key devices suitable for use in next-generation technologies such as displays, power devices, and artificial intelligence devices such as neural networks.

## Methods

### Samples

The fabricated GTO TFTs have a structure with a bottom gate and top contact. We used the same structure as that of previously reported IGZO TFTs^36^ in order to evaluate the compatibility of GTO with the other oxide semiconductor materials and compare their performance under the same conditions. The GTO active layers were deposited by RF magnetron sputtering using a sintered GTO ceramic target (99.99%, Ga:Sn = 1:3 in atomic ratio). The vacuum chamber was evacuated to 1 × 10^−4^ Pa and the sputtering gas pressure was controlled using a vacuum valve to introduce Ar and O_2_ gas into the chamber at a fixed flow rate of Ar:O_2_ = 20:1 sccm determined by a mass flow controller. The GTO was deposited on Si wafers (p-type; 0.01 Ω·cm; 50 mm in diameter) for use as gate electrodes in the TFT which had a 150 nm thermal oxide (SiO_2_) layer as the gate insulator. Source and drain (S/D) electrodes of Ti (20 nm in thickness) and Au (20 nm in thickness), respectively, were deposited using thermal evaporation on the GTO active layer. Both the semiconductor layer and S/D electrodes were patterned using stainless steel shadow masks set on top of the substrates. Post annealing was performed in air at 350 °C for 1 h using an annealing furnace. No passivation layer was deposited on the back channel of the TFTs^36^ to avoid undesired effects from plasma processes such as CVD for the fabrication of passivation films and dry etching processes^43^.

### Characterisation

Electrical properties such as *I*_ds_-*V*_gs_ and *I*_ds_-*V*_ds_ of GTO TFTs were measured in air at around 25 °C using a semiconductor parameter analyser (Agilent 4156 C). TFT parameters such as *μ*_FE_, *S* factors, and *V*_th_ were calculated from TFT theory, such as *I*_ds  _= *μ*_FE_ *c*_*i*_(*W/L*)(*V*_gs_ − *V*_th_)*V*_ds_, where, *c*_*i*_ is the capacitance of the TFT of unit area (F/cm^2^), *W/L* is the ratio of the channel width to the channel length defined between the S/D electrodes. The stability of the GTO TFTs was evaluated under accelerated operating conditions of devices, including voltage, temperature, and light illumination stresses using conditions of *V*_g  _ = 20 V/ −20 V for positive or negative bias, respectively, 60 °C, and irradiation from an white LED lamp with wavelength from 450 nm to 780 nm. By combining these conditions, we evaluated the stability of the material under positive bias stress (PBS), positive bias temperature stress (PBTS), positive bias illumination stress (PBIS), negative bias stress (NBS), negative bias temperature stress (NBTS), and negative bias illumination stress (NBIS). The optical absorbance (*A* %) was obtained from the transmittance (*T* %) and reflectance (*R* %) using A = (100 − (*R* + *T*)), measured using a spectrometer from the ultraviolet to visible range (UV-VR) (300 nm to 800 nm). XRD pattern in 2*θ/θ* scan of GTO thin film deposited on a quartz substrate was measured with Rigaku SmartLab X-ray diffractometer (Cu Kα radiation).

## Additional Information

**How to cite this article:** Matsuda, T. *et al*. Rare-metal-free high-performance Ga-Sn-O thin film transistor. *Sci. Rep.* **7**, 44326; doi: 10.1038/srep44326 (2017).

**Publisher's note:** Springer Nature remains neutral with regard to jurisdictional claims in published maps and institutional affiliations.
